# Black silicon with self-cleaning surface prepared by wetting processes

**DOI:** 10.1186/1556-276X-8-351

**Published:** 2013-08-13

**Authors:** Ting Zhang, Peng Zhang, Shibin Li, Wei Li, Zhiming Wu, Yadong Jiang

**Affiliations:** 1 State Key Laboratory of Electronic Thin Films and Integrated Devices, School of Optoelectronic Information, University of Electronic Science and Technology of China (UESTC), Chengdu 610054, People's Republic of China

**Keywords:** Black silicon, Metal-assisted wet etching, Hydrophobic surface, Reflectance, Absorption

## Abstract

This paper reports on a simple method to prepare a hydrophobic surface on black silicon, which is fabricated by metal-assisted wet etching. To increase the reaction rate, the reaction device was placed in a heat collection-constant temperature type magnetic stirrer and set at room temperature. It was demonstrated that the micro- and nanoscale spikes on the black silicon made the surface become hydrophobic. As the reaction rate increases, the surface hydrophobicity becomes more outstanding and presents self-cleaning until the very end. The reflectance of the black silicon is drastically suppressed over a broad spectral range due to the unique geometry, which is effective for the enhancement of absorption.

## Background

Black silicon has attracted wide attention due to its extremely low reflectivity (even below 1%) since a nanostructured silicon surface was built by femtosecond laser pulse irradiation in 1999 [1]. Owing to its promising future, extensive research has been done to create random nanospikes or nanopores on silicon surface by different approaches, for instance, femtosecond laser pulse irradiation [1,2], metal-assisted wet etching [3-5], reactive ion etching [6,7], and electrochemical etching [8]. After surface modification on silicon wafer, efficient suppression of reflection in a broad visible spectral range can be achieved through multiple reflection and absorption. Branz et al. [9] proposed that a network of nanopores prepared by Au-assisted wet etching formed the density-grade layer between the air-nanopore interface and the nanopore-silicon interface, which can reduce reflectance at wavelengths from 300 to 1,000 nm to below 2%. Along with grade depth increases, reflectivity decreases exponentially. Especially in the gradient depth of approximately 1/8 the vacuum wavelength or half the wavelength in silicon, the exponential decline is significant.

The surface hydrophobicity of black silicon has a potential application in addition to the abovementioned excellent features, taking advantage of the feature that high technologies have been successfully developed, for instance, nanodome solar cells with anti-dust surface [10]. Due to chemical etching, the surface energy is reduced [11] and the surface geometry is reconstructed [12]. Both sides will be conducive to the enhancement of intrinsic hydrophobic surface. Local surface roughness is considered relevant to surface hydrophobicity [13]. We can use different chemical and physical approaches, such as nanocoating materials [14], femtosecond laser irradiation [15], photolithography [16,17], etc., to modify surfaces, leading to the enhancement of surface hydrophobicity. Usually, these methods are complicated. In this paper, we report a hydrophobic property of black silicon surface. The micro- and nanospikes are prepared by metal-assisted wet chemical etching, without any complex nanomaterial coating deposition.

## Methods

N-type single-crystal silicon wafers (100) with a resistivity of 6 to 8 Ω cm were cleaned by RCA standard cleaning procedure with each step for 15 min. After cleaning, the wafers were etched with HF in order to remove the unwanted native oxide layer. In the following step, the wafers were etched in a mixed solution containing H_2_O_2_, C_2_H_5_OH, H_2_O, HF, and HAuCl_4_ with a typical ratio of 10:4:4:2:1, resulting in pores. This treatment occurred at room temperature for 8 min.

As a control, one beaker (marked as A) was placed in a digital constant temperature water bath (HH-2, Guohua Electric Devices, Changzhou, China) and set at room temperature. The other (marked as B) was laid in a heat collection-constant temperature type magnetic stirrer (HCCT-MS; DF-101S, Wuhan, Sensedawn Science &Technology, Wuhan, China) at the same temperature. The samples in the beakers were correspondingly signed as A and B.

The morphology of the textured silicon was characterized using a scanning electron microscope (SEM; JSM-5900 Lv, JEOL, Tokyo, Japan). An atomic force microscope (AFM; SPA-400 SPM UNIT, DAE HWA NI Tech, Pyeongtaek-si, South Korea) was used to characterize the topology of the black silicon in tapping mode. A UV-visible-near-infrared (UV–vis-NIR) spectrophotometer (UV-3600, Shimadzu, Tokyo, Japan) with an integrating sphere detector was used to measure the total (specular and diffuse) reflectance (*R*) and transmittance (*T*). The static contact angles (CAs) were measured by capturing images of deionized water droplets using a drop shape analysis system, referred to as a sessile drop method. With a software equipped with an optical contact angle measuring instrument (OCAH200, Data Physics Instruments, Filderstadt, Germany), the CA values between the tangent of the drop and the horizontal plane at the point of contact with the black silicon surface were calculated. The mean value was calculated from at least four individual measurements, and each individual measurement contains independent values of the left and right contact angles.

## Results and discussion

In the metal-assisted chemical etching procedure, the Si substrate is subjected to an etchant, which is composed of HF and H_2_O_2_ compound. As a consequence, the nanoscale noble metal particles sink into the Si substrate, resulting in pores. It was found that the initial morphology of the noble metal coverage is crucial to the generation of the unique geometries of Si substrate [18]. During metal-assisted chemical etching, the noble metal adheres to the silicon surface and acts as a cathode to reduce the oxidant H_2_O_2_ generating holes (*h*^+^). Then the holes are poured into the valence band of silicon to oxidize and dissolve the Si substrate in the HF solution. Where the cathode reaction can be written as H_2_O_2_ + 2H^+^ → 2H_2_O + 2*h*^+^, at the anode (silicon substrate), the reaction is $\mathrm{Si}\;+\;6\mathrm{HF}\;+\;nh^{+}\;\to \;nH^{+}\;+\;H_{2}{\mathrm{SiF}}_{6}+\;\frac{4-n}{2}H_{2}↑$[19]. So the overall reaction is $\mathrm{Si}\;+\;\frac{n}{2}H_{2}O_{2}+\;6\mathrm{HF}\;\to \;nH_{2}O\;+\;H_{2}{\mathrm{SiF}}_{6}\;+\;\frac{4-n}{2}H_{2}↑$. When Au is used as a catalyst, the reaction of metal-assisted chemical etching of silicon in a solution of HF and H_2_O_2_ is $\mathrm{Si}+\;H_{2}O_{2}\;+\;6\mathrm{HF}\;\overset{\mathrm{Au}}{\to }\;{2H}_{2}O\;+\;H_{2}{\mathrm{SiF}}_{6}\;+\;H_{2}↑$. Details on the cathode and anode reaction mechanism of the metal-assisted chemical etching can be found elsewhere [18,20].

In an effort to comprehend the mechanism of the formation of pores, the following statements about isotropic etching give a better understanding. The etching process continues as the catalysis of Au nanoparticles, which are merely from the reduction of HAuCl_4_ by H_2_O_2_. In the etching solution, Au particles adhere to the wafer surface via diffusion. Due to the electromotive force of Au particles being higher than that of silicon, this will form the local electromotive difference of potential. After the beginning of etching, nanopores are formed on the wafer surface, and as this process continues, the Au nanoparticles will subside to the bottom of the nanopores to ensure bottom etching. There is not enough energy to make a hole reach the surfaces of the sidewall because the sidewall of the nanopores are far away from the Au nanoparticles, so the lateral etching will stop. The above process results in the formation of nanopores.

**Figure 1:**
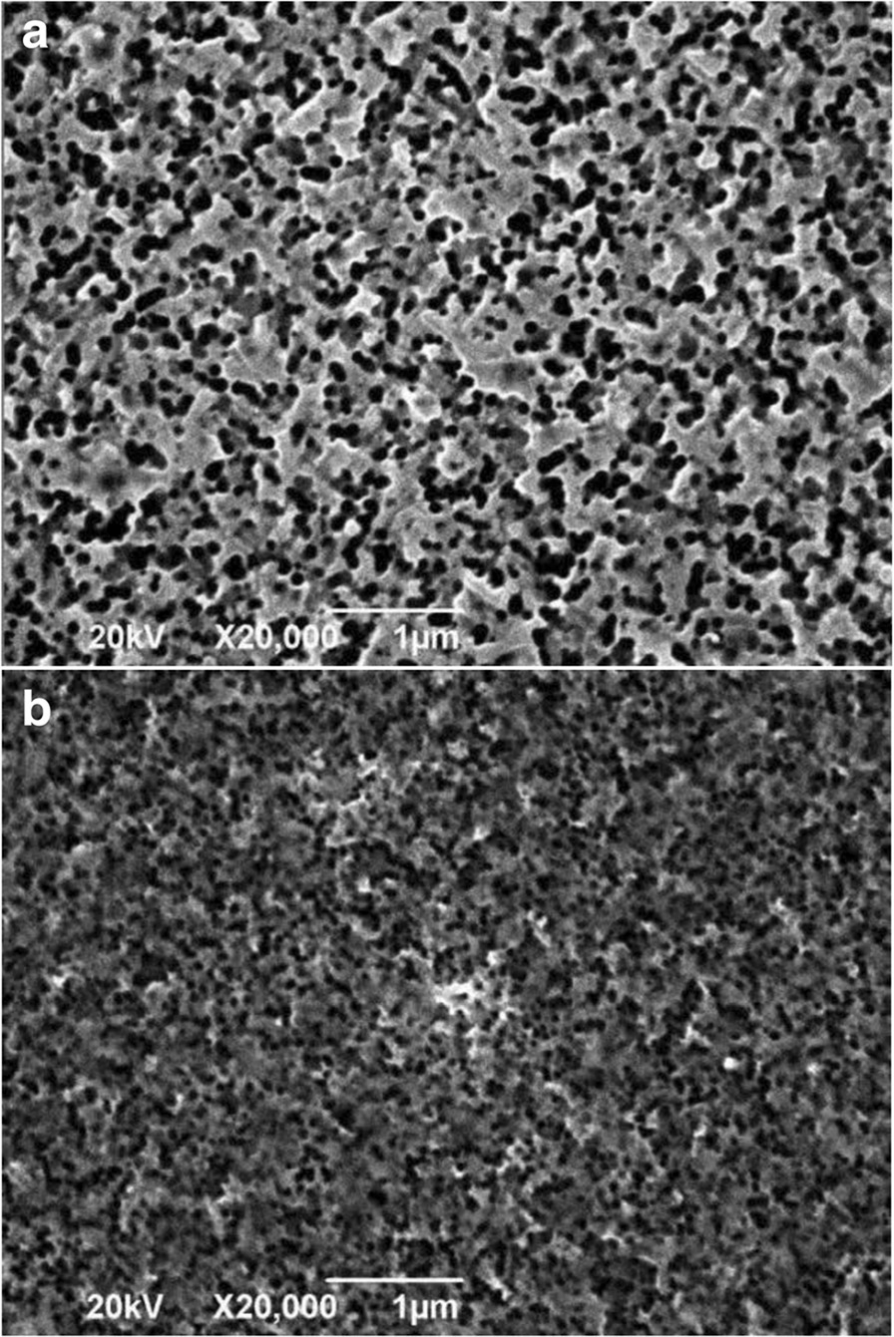
**Top view of the black silicon produced by metal**-**assisted chemical wet etching. (a)** Sample A in the digital constant temperature water bath. **(b)** Sample B in the heat collection-constant temperature type magnetic stirrer.

**Figure 2:**
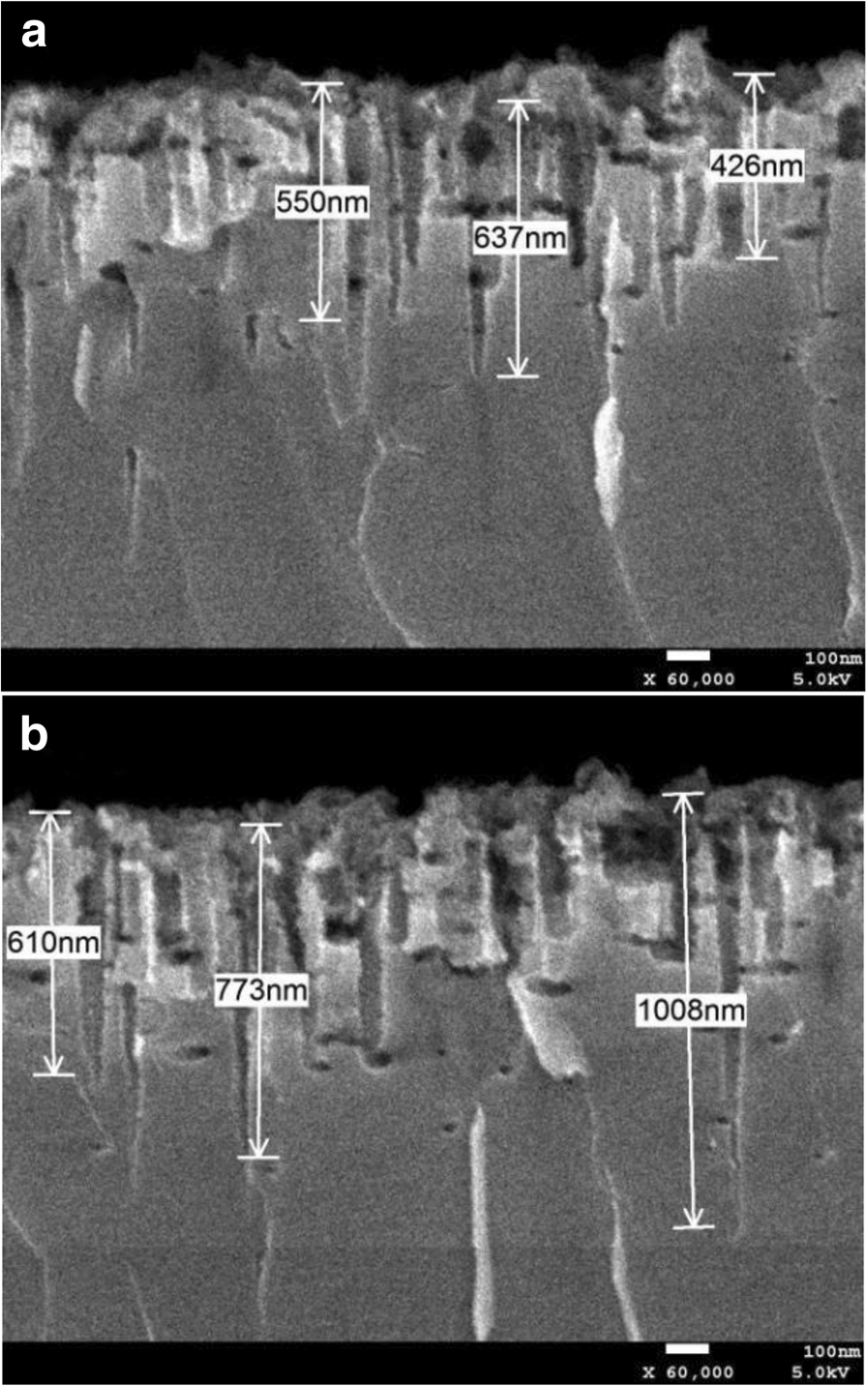
**Cross section of the black silicon produced by metal**-**assisted chemical wet etching. (a)** Sample A in the digital constant temperature water bath. **(b)** Sample B in the heat collection-constant temperature type magnetic stirrer.

The procedure of etching with a color change on the silicon wafer from gray to complete black is observed obviously. From the SEM images (Figures 1 and 2), the existence of nanoscale pores and spikes is seen. The nanopores shown in Figure 1b are more uniform and smaller than those shown in Figure 1a, and the length of the nanospikes in Figure 2b is much longer than that in Figure 2a.

**Figure 3:**
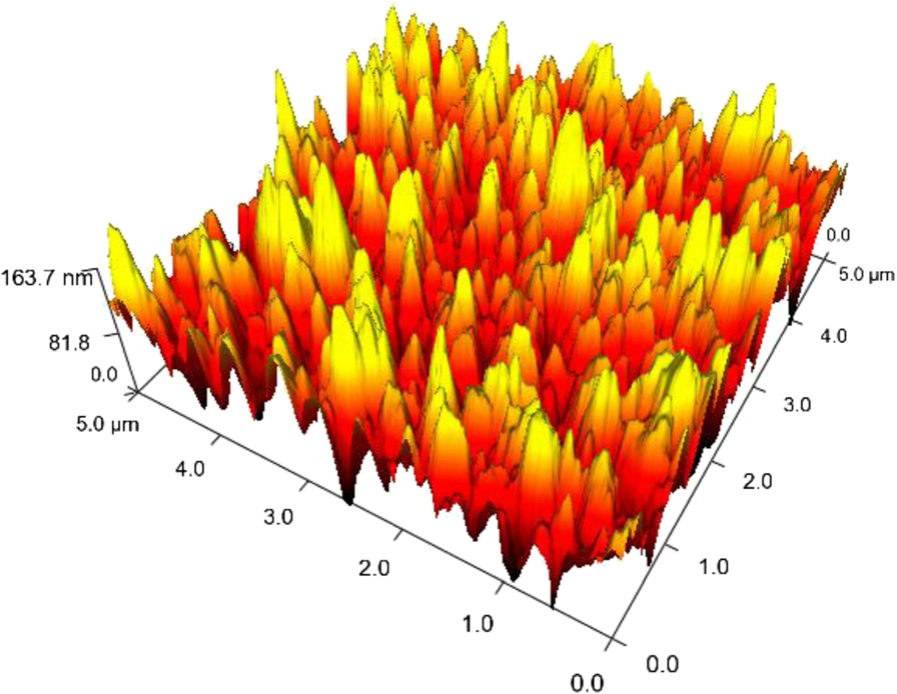
**3D topological AFM image (5 × 5 μm**^**2**^**) of sample B.**

When the two samples were taken out simultaneously from the two beakers, only sample B in the HCCT-MS showed clear hydrophobicity. The mixing process accelerates the whole chemical reaction; nanospike structures are clustered together at the point. In Figure 3, the three-dimensional (3D) topological AFM image (5×5 μm^2^) can reasonably support the above hypothesis. The nanoscale structures together with the few microscale features decorating the spikes result in a pronounced increase of the overall roughness. The increase of local surface roughness is beneficial for the enhancement of surface hydrophobicity. It is assumed that the surface of sample B prepared with this procedure possesses the hydrophobic self-cleaning function due to the second length scale morphology. It is well known that a hydrophobic surface generally refers to a surface with a water contact angle larger than 90°. When a surface has a water contact angle larger than 150°, it is called a superhydrophobic surface.

The initial understanding on a superhydrophobic surface is mainly from lotus leaves [21], which consist of micro- and nanostructures with self-cleaning capability by instinct. In nature, it is very common that a hydrophobic surface is obtained from the self-cleaning phenomenon. For instance, the Compositae petal leaves with a water contact angle of 128° shows a hydrophobic self-cleaning function. In this paper, the silicon wafer has been modified with metal-assisted wet etching. After modification, the water contact angle on the surface of black silicon clustered by nanospike and few microspike structures is adequate to achieve self-cleaning. According to the experimental measurement, the mean static contact angle of sample B is approximately 118°, while that of sample A is approximately 82°. The textured silicon (sample B) with a dualistic structure can imitate Compositae petal leaves ideally.

The water contact angles in such cases may be interpreted by describing the Cassie-Baxter wetting state, where liquid drops do not completely penetrate the nanostructures and air pockets are trapped inside the spikes underneath the liquid drop [22-24]. A relationship that describes the contact angle on the textured surface is expressed by the equation cos *θ*^CB^ = *f* cos *θ* + *f* − 1, where *θ*^CB^ is the liquid–solid contact angle on the textured surface, *θ* is the static contact angle on the flat surface, and *f* is the fraction of the liquid–solid contact area.

Therefore, depending on the value of the *f* factor, the surface can be either hydrophilic or hydrophobic. According to the above equation, the smaller the value of *f*, the higher the increase of the contact angle. So it is essential to make a smaller contact area in order to obtain the higher contact angle. For example, the surface hydrophobicity can be improved in the preparation of a nanostructured silicon section. The result is consistent with the reports that black silicon was obtained by a photochemical procedure based on anisotropic etching [25].

**Figure 4:**
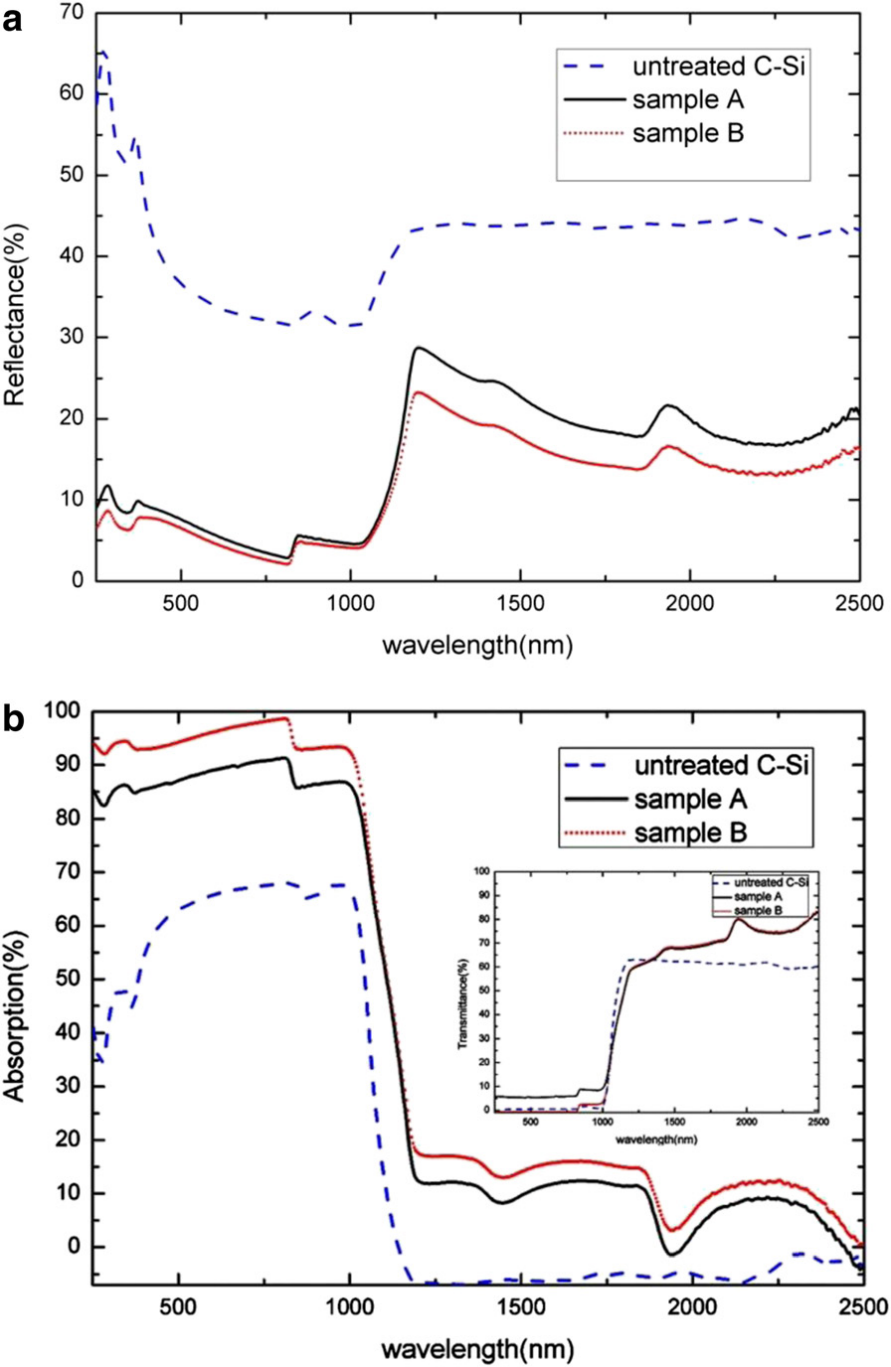
**Total reflectance and absorption spectra. (a)** Total reflectance spectra and **(b)** total absorption spectra for the A, B, and untreated C-Si samples with wavelength ranging between UV and NIR. The inset shows total transmittance spectra for both treated and untreated samples.

The total optical reflectance spectra of A, B, and untreated crystalline silicon(C-Si) samples were measured for normal incident covering the spectral regions above and below the bandgap of silicon with a spectrophotometer and an integrating sphere detector. As shown in Figure 4a, the reflectance spectrum of the untreated sample (blue dashed line) shows the typical high reflectivity as expected, while the reflectance of samples A and B was drastically suppressed over the spectrum from the UV to the near IR. It is worthwhile to note that the reflectivity of sample B (red line) is 10% lower than that of sample A (black line). The reflectivity of sample B also increases evidently (23%) beginning from the wavelength of approximately 1,216 nm.

**Figure 5:**
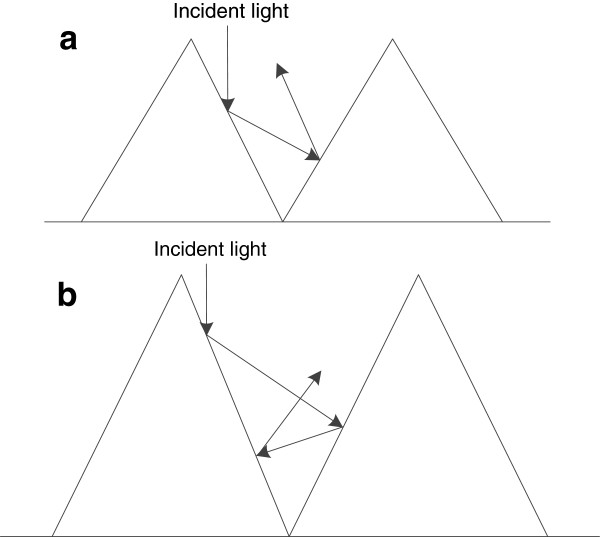
**Optical path of incident light on the black silicon spike structures. (a)** Sample A in the digital constant temperature water bath. **(b)** Sample B in the heat collection-constant temperature type magnetic stirrer.

The absorption curves of the textured samples in Figure 4b, calculated by the formula *A*=1 − *R* − *T*, also show a stronger absorption than the untreated sample over a broad spectral range. Obviously, the absorption of sample B is strongest in the range of 250 to approximately 1,100 nm. Over the UV–vis spectrum, the absorption of sample B is above 90%, even up to 98%. It is noteworthy that the decrease of reflectance below the bandgap is not accompanied by the increase of absorption, instead of the increased transmittance (as shown in the inset). Both textured and untreated silicon are transparent above the wavelength of 1,100nm. It is more important that the total reflectance and absorption of sample B at the wavelength of approximately 1,100 nm are approximately 8.649% and 54.32%, respectively, and the results compared to those of sample A are higher. By the same token, the appearance of random microscale spikes can enhance optical absorption inside the material. This behavior can be reasonably explained by multiple scattering effects with second length scale arrays. As shown in Figure 5, the length of spikes in Figure 5b is longer than that in Figure 5a, so the frequencies of reflectance in Figure 5b are more. So the more frequencies of reflectance are, more light can be trapped and higher absorption is obtained.

Once black silicon materials are used on solar cells or photovoltaic detectors, dust particles accumulating on the device architectures will seriously imprison sunlight and eventually lead to the reduction of device efficiency and device life. Devices with self-cleaning function can easily avoid the abovementioned problem. It is important that we use simple chemical etching to achieve the self-cleaning function of black silicon surface. It paves the way for our further study on the morphology and topology of textured silicon by chemical etching.

## Conclusions

In conclusion, we have demonstrated the self-cleaning function of black silicon surface which was textured by metal-assisted chemical wet etching. SEM and AFM images confirmed that the black silicon surface textured in the HCCT-MS had both micro- and nanoscale structures. The static contact angle of approximately 118° is adequate to make the surface hydrophobic with a self-cleaning performance. The reflectance of sample B is suppressed due to the unique geometry, which is effective for the enhancement of absorption. How to make better use of the feature in a specific environment still requires further study. The novel construction of a hydrophobic surface on black silicon wafer may be applicable to various applications.

## Competing interests

The authors declare that they have no competing interests.

## Authors’ contributions

TZ and PZ designed and carried out the experiments. TZ, PZ, SL, and ZW participated in the work to analyze the data and prepared the manuscript initially. SL, WL, ZW, and YJ gave equipment support. All authors read and approved the final manuscript.
